# 

**Published:** 1860-07

**Authors:** 


### INDEX.

PAGE.

A New Method of Using Vulcanite—By P. G. C. Hunt......... 12
Associations......................... 55
Artificial Dentures—By Dr. J. Allen....................................134
American Dental Convention.....143
A Critic Criticized...................218
Anæsthetics in Hospital Practice.281
American Dental Association,...182
" " " 244
American Dental Convention,....181
" " " 247
A Singular Specimen................118
Advice to Patients..................... 50
Acute Periostitus......................292
Artificial Dentures—By John Allen .................................... 40
Adventure with the File........... 48
A Rare Form of Fracture of the Lower Jaw treated by a New Method............................285

Biographical Sketch of Dr. Alvin Blakesley, of Utica, N. Y....275
Buccal Secretions—By J. Taft....121
Buccal Inflammations ............. 36
Bicuspid Pivot Teeth................117
Bloodless operation for Cleft Palate....................................296

Correspondence.............32, 94, 306
Compatability—By J. Taft......... 82
Cincinnati Dental Association.... 85
Case of Abscess of Parotid Gland —Lykins............................ 44
Care of the Sick....................... 54
Cincinnati Dental Association.... 55

Dental Surgery — By P. G. C. Hunt..... ....................... 5
Discussions of the Kentucky State Dental Association,............ 19
Dental Teaching—By W. H. Atkinson ...........................185
Death from Inhalation of Chloroform..........................297, 303

PAGE.

Defective Assimilation in Infants294
Duties of Chemists...................248
Development of the Teeth of Cattle...........................................239
Dental Fistulas......................... 38
Dental Meetings........................ 60

Editorial...60, 117, 244, 181, 308, 367
Extortion.................................256
Enlargement of Submaxillary Glands, after removal of Cancer of the Lip.....................112
Extreme Congenital Deformity...287
Ether and Chloroform...............289

Fang Filling—By A. A. Blount, M. D .................................. 70
Filling Teeth—By Dr. H. A. Smith 75
Fatality from Chloroform..........295
Fang Filling and Treatment of the Alveolar Abscess—By Dr. B. A. Rose.................. 87

Gutta Percha for Separating and Regulating Teeth — By B. Wood.................................... 5

Hypertrophy...........................267

Lord Oxygen—By Geo. Watt.....321
Language— Mistakes— Corrections.................................... 16

Mad River Valley Dental Society 89
Mallet-ing...............................142
Mounting Teeth with Double Plates .... .........................183
Modifying Influence of Caries....308

New Fusible Alloys — By B. Wood, M. D...................... 1
New Facts in Regard to Gold.....180
New Application of Chloroform in Neuralgia......................241
New York Dental Institute........310

PAGE.
Proceedings of Societies..19, 278, 358
Proceedings of the Western Dental Association.................. 91
Proceedings of the American Dental Association...........222
Purple of Cassius—By Prof. Wm. Calvert, D. D. S................254
Patent Instruments—By J. A. Murphy, M. D.......... ........351
Patents, remarks on—By T.......353

Removal of the Lower Jaw for Osteo-Sarcoma, of immense size—By Geo. C. Blackman, M. D..............................356

Selections...36, 97, 180, 237, 281, 362
Selection and arrangement of Teeth in Constructing Dental Substitutes—By Prof. W. Calvert, D. D. S................. 65
Sensitive Dentine — By Geo. F. Foote..............................129
Slabs from the Furnace—By Joe. Stackpole .......................213

PAGE.
Treatment of a Diseased Tooth—By J. T......................... 80
The Anatomy, Physiology, Pathology and Remedial Treatment of the Fifth Pair of Nerves—By J. H. McQuillen, D. D. S.........189, 259, 330
The Oneness of Dentistry,.........210
The Province of the Dentist—By J. Taft.........................249, 313
Thoughts on Dental Caries—By Geo. Watt .......................270
Treating and Capping Exposed Nerves—By W. A. Pease.....316
Transactions of the American Dental Association............312
Treatment of Neuralgia, by Subcutaneous injection ...........101
Trip to Washington ................ 63
Turkish Bath ..........................231
The Mallet—Rubber Work.........245

Use and Abuse of Tobacco.........298

Vulcanite Ideas........................ 18
Ventilation of Rooms at Night...184

VOLUME XIV.

### DENTAL REGISTER,

### OF THE WEST.

This volume will be issued in 6 months, commencing with the July Number, and closing with the December Number.

The price is **ONE DOLLAR**, for this volume.

**NOW IS THE TIME TO SUBSCRIBE.**

*Send on your Dollar and have your name enrolled.*

**TERMS - - STRICTLY CASH, IN ADVANCE.**

A FEW ADVERTISEMENTS WILL BE INSERTED AS FOLLOWS.

| | |
|---|---|
| One Page, - - - - - | $30. |
| Half Page, - - - - | $20. |
| Quarter Page, - - - - | $15. |

### CONTENTS.

ORIGINAL ESSAYS AND COMMUNICATIONS.

New Fusible Alloys. By B. Wood, M. D. .... 1

Dental Surgery. By P. G. C. Hunt .... 5

Gutta Percha for Separating and Regulating Teeth. By B. Wood .... 10

A New Method of using Vulcanite. By P. G. C. Hunt .... 12

Language—Mistakes—Corrections .... 16

Vulcanite Ideas .... 18

PROCEEDINGS OF SOCIETIES.

Discussions of the Kentucky State Dental Association .... 19

Correspondence .... 32

Selections .... 36

Associations .... 55

